# Effects of Altered Catecholamine Metabolism on Pigmentation and Physical Properties of Sclerotized Regions in the Silkworm Melanism Mutant

**DOI:** 10.1371/journal.pone.0042968

**Published:** 2012-08-24

**Authors:** Liang Qiao, Yuanhao Li, Gao Xiong, Xiaofan Liu, Songzhen He, Xiaoling Tong, Songyuan Wu, Hai Hu, Rixin Wang, Hongwei Hu, Lushi Chen, Li Zhang, Jie Wu, Fangyin Dai, Cheng Lu, Zhonghuai Xiang

**Affiliations:** 1 State Key Laboratory of Silkworm Genome Biology, Southwest University, Institute of Sericulture and Systems Biology, Southwest University, Chongqing, China; 2 Institute of Agriculture and Life Science, Chongqing University, Chongqing, China; 3 College of Biotechnology, Southwest University, Chongqing, China; University of Kentucky, United States of America

## Abstract

Catecholamine metabolism plays an important role in the determination of insect body color and cuticle sclerotization. To date, limited research has focused on these processes in silkworm. In the current study, we analyzed the interactions between catecholamines and melanin genes and their effects on the pigmentation patterns and physical properties of sclerotized regions in silkworm, using the melanic mutant *melanism* (*mln*) silkworm strain as a model. Injection of β-alanine into *mln* mutant silkworm induced a change in catecholamine metabolism and turned its body color yellow. Further investigation of the catecholamine content and expression levels of the corresponding melanin genes from different developmental stages of Dazao-*mln* (mutant) and Dazao (wild-type) silkworm revealed that at the larval and adult stages, the expression patterns of melanin genes precipitated dopamine accumulation corresponding to functional loss of *Bm-iAANAT*, a repressive effect of excess NBAD on *ebony*, and upregulation of *tan* in the Dazao-*mln* strain. During the early pupal stage, dopamine did not accumulate in Dazao-*mln*, since upregulation of *ebony* and *black* genes led to conversion of high amounts of dopamine into NBAD, resulting in deep yellow cuticles. Scanning electron microscope analysis of a cross-section of adult dorsal plates from both wild-type and mutant silkworm disclosed the formation of different layers in Dazao-*mln* owing to lack of NADA, compared to even and dense layers in Dazao. Analysis of the mechanical properties of the anterior wings revealed higher storage modulus and lower loss tangent in Dazao-*mln*, which was closely associated with the altered catecholamine metabolism in the mutant strain. Based on these findings, we conclude that catecholamine metabolism is crucial for the color pattern and physical properties of cuticles in silkworm. Our results should provide a significant contribution to Lepidoptera cuticle tanning research.

## Introduction

Diversified body color and sclerotized exoskeleton are essential for the survival and evolution of insects, and catecholamines play an important role in the pigmentation and sclerotization of cuticles [1], [2], [3], [4], [5], [6]. In the melanin metabolism pathway of insects, tyrosine is converted into four types of catecholamines, specifically, dopa, dopamine, N-acetyl dopamine (NADA) and N-β-alanyldopamine (NBAD), through a series of catalytic reactions, such as hydroxylation, decarboxylation, and addition of acetyl or β-alanyl groups [3], [4], [5], [6], [7], [8]. After oxidation by phenoloxidases, such as Laccase2, these catecholamines participate in insect cuticle tanning [9], [10], [11], [12].

Varying levels of catecholamines lead to different body colors in insects [13], [14], [15], [16], [17]. Simultaneously, expression levels of melanin metabolism genes influence the synthesis of catecholamines that contribute to insect body color [18], [19], [20]. Additionally, different pigment precursors can be produced by diverse genes or vary according to the expression levels of these genes, resulting in distinct pigment patterns in insects [21], [22], [23], [24].

Newly formed cuticles of insects are usually soft and pale [3], [25]. During the tanning process, dopamine, NBAD and NADA are transported by epidermal cells, and the catecholamines are subsequently converted into methylated or unmethylated quinones through oxidation. The resulting chemical compounds generated are cross-linked with cuticle proteins and mixed with the cuticular matrix, which stabilizes the exoskeleton of insects and causes it to harden [3], [5], [26], [27]. The mixing process can occur at different developmental stages, termed pre-ecdysial sclerotization, post-ecdysial sclerotization and puparium formation [3]. Variable catecholamine types and contents are required in different regions during tanning [17], [18], [28], [29], [30]. Consistently, another study reported that different catecholamine contents are required during tanning at various developmental stages [31]. Since catecholamines participate in the insect cuticle tanning process, metabolism of these compounds is critical in determining the physical properties of the cuticle and exoskeleton in insects, including their structures [32], [33], hardness and mechanical properties [18], [34], [35].

The silkworm melanin synthesis pathway has been predominantly characterized through research on body color mutants [20], [23], [36], [37], [38], [39] (Figure S1). Catecholamines and melanin genes have been shown to have a critical impact on color patterns in silkworm. We confirmed that the *melanism* mutation (*mln*) is caused by dopamine over-accumulation resulting from mutation of the *Bm-iAANAT* gene (insect arylalkylamine-N-acetyl transferase gene of silkworm), and the melanic phenotype is exhibited at the larval, late pupal and adult stages [37]. However, the impacts of mutual regulatory interactions between catecholamines and their corresponding genes on pigmentation and physical properties of sclerotized regions are unclear at present. In the current study, catecholamine metabolism in the *mln* mutant was altered via injection of β-alanine, which turned the body color lighter and more yellow. Moreover, we systematically investigated the differences in contents of the four catecholamines and expression levels of the respective melanin metabolism genes between wild-type and mutant strains at different developmental stages. Cross-sectional differences in the adult dorsal plates and dissimilarities in the mechanical properties of wings between the mutant and wild-type groups were analyzed. Based on the results, we conclude that the catecholamine content in the mutant strain is dependent on regulation of the corresponding melanin synthesis genes. Additionally, catecholamines appear crucial in determining the unique pigment pattern of the mutant strain, and affect construction of the exoskeleton and mechanical properties of wings. This rare mutant silkworm may thus serve as a model for evaluating the relationship between the content and make-up of catecholamines in insects and expression levels of their respective genes, and provide further information regarding the influence of catecholamines on pigmentation, construction and mechanical properties of Lepidoptera cuticle, yielding valuable insights into the mechanisms underlying Lepidoptera cuticle tanning.

## Materials and Methods

### Silkworm strains

The *melanism* mutant strain, Dazao-*mln* (near isogenic line of *mln*, backcrossed for 24 generations), and wild-type Dazao were supplied by the silkworm gene bank at Southwest University. Insects were reared on fresh mulberry at 25°C, and subjected to a regimen of L12∶D12.

### Chemicals

Dopamine (H8502-5G), dopa (D9628-5G) and β-alanine (A9920-100G) were purchased from Sigma. NBAD and NADA standards were provided, courtesy of Professor Michael R. Kanost, Dr. Neal T. Dittmer (Department of Biochemistry, 141 Chalmers Hall, Kansas State University, Manhattan, KS 66506-0116, USA) and Professor Manickam Sugumaran (Department of Biology, University of Massachusetts, Boston 100 Morrissey Blvd Boston, MA 02125).

### Real Time RT-PCR

To determine the differences in dopamine metabolism-related genes between mutant and wild-type strains, total RNA was extracted using TRIzol from the heads of fifth instar larvae (immediately and 12 h after the fourth molt), anterior wings of adults, pupae at two, four, six and eight days after the beginning of pupation, and whole adults, followed by reverse transcription to cDNA. Quantitative RT-PCR was performed using the ABI Prism 7000 sequence detection system (Applied Biosystems), according to the manufacturer's instructions. The primers for *BmDdc* (dopa decarboxylase gene, AF372836.1), *Bmebony* (N-β-alanyl dopamine synthetase gene, NM_001145321.1), *Bmblack* (aspartate decarboxylase gene, unpublished data), *Bmtan* (N-β-alanyl dopamine hydrolysis enzyme gene, NM_001177411) and *BmLaccase2* (NM_001109925) are listed in Table S1.

### Analysis of the catecholamine contents in *mln* mutant and wild-type strains during various developmental stages

Heads of instar larvae immediately after the fourth molt, 12 h fifth instars, day 2 of pupation, and moths from the wild-type and mutant strains were selected. Catecholamines were extracted and determined according to Koch's method [40]. Shimadzu LC20A and Symmetry Shield RP18 (5 µm, 4.6×250 mm, Waters) columns were used for high-performance liquid chromatography (HPLC) analysis. The column flow rate was 0.8 ml/min. Triple biological repeat were carried out for each sample, consisting of no less than three tissues or individuals (for each sample, 25 heads, four pupae and four moths were used). The four types of catecholamines were identified based on retention times, compared to known standards, as follows: dopa, 5.857 min; dopamine, 8.228 min; NBAD, 18.765 min, and NADA, 24.020 min (Figure S2).

### Injection of β-alanine and catecholamines

After 6 days of pupation, mutant pupae were selected for β-alanine injection. The injection dose gradient was as follows: 300 µg, 500 µg and 700 µg/pupa. Evaluation of phenotypic characteristics, quantitation of catecholamines, and expression level analysis of the corresponding melanin metabolism genes were performed after eclosion. Mutant *mln* pupae injected with 0.75% saline were used as the control. The dopamine injection groups were as follows: injection with 500 µg dopamine on day 2 of pupation followed by observation on day 3, injection with 500 µg dopamine on day 4 of pupation followed by observation on day 5, and injection with 1 mg of dopamine on day 6 of pupation followed by phenotype observation and investigation of changes in the expression levels of melanin genes on day 7 of pupation. We used 0.75% saline-injected individuals as the control. For the NBAD and NADA groups, *mln* pupae were injected with 500 µg of NBAD or NADA on day 6, with 100 mM HCl (solvent) as control, and used on day 7 for phenotype and melanin gene expression analyses.

### Scanning electron microscopy analysis

Adult dorsal plates of wild-type, *mln*, *mln* injected with NBAD and *mln* injected with NADA were digested using 1 mg/ml proteinase at 37°C for 20 min to remove attached muscle tissue, embedded, and sliced with a Leica Frozen Section Machine (CM1900). Subsequently, sections were thoroughly rinsed with ultrapure water and subjected to a series of dehydration steps, according to Hu's method [41]. Dorsal plates were sputter-coated with gold using a Hitachi E-1010 high-resolution sputter coater. Dorsal plates from both strains of silkmoth were investigated under a Hitachi S-3000N Scanning electron microscope (Hitachi, Tokyo, Japan).

### Mechanical properties of adult wings

The mechanical properties of adult wing were investigated using a DMAQ800 Dynamic Mechanical Analyzer. Same-sized male moths from the wild-type and mutant groups were incubated at room temperature for 6 h after eclosion to ensure their wings were fully tanned, and placed in a −20°C environment for 20 min. Anterior wings were removed, trimmed into a 1 cm×0.6 cm rectangle shape to verify that the materials tested were of a similar size, and sourced from the same part of the wing. Wing thickness was measured using electronic vernier calipers. Materials were incubated at 25°C and 75% relative humidity for 2 h before mechanical testing, and fixed between the two grips of the analyzer. Storage modulus (E′), loss modulus (E″) and loss tangent (tanδ = E′/E″) were determined using the film-stretching mode at 0.1% strain (at this level of strain, the wings would not be torn apart) and scanned using frequencies ranging from 100 Hz to 0.1 Hz.

## Results

### Phenotype of the *mln* mutant pupae after excess dopamine injection

**Figure 1:**
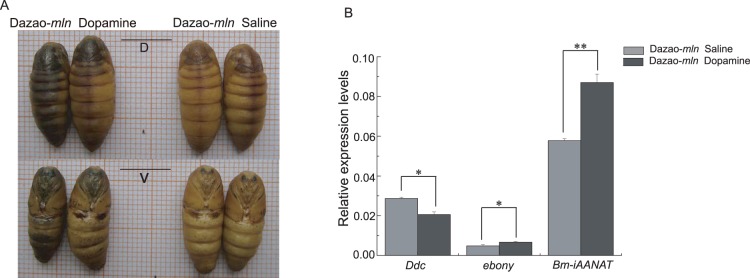
Phenotype of *mln* pupae after dopamine injection and expression levels of the corresponding melanin genes. (A). Phenotype after dopamine injection (injected on day 6 and examined on day 7 of pupation). D represents dorsal side, V represents ventral side. bar: 1 cm. (B). Expression levels of the corresponding melanin genes. (Student's t-test, n = 3. *p<0.05, **p<0.01, data represent mean±S.D.).

We injected excess dopamine into *mln* pupae on day 6 of pupation, and examined their phenotypes on day 7. The injected group was evidently melanized, compared with the control group (pupae became more melanized with time), indicative of accumulation of large amounts of melanin in injected individuals (Figure 1A). Investigation of the expression levels of the corresponding melanin genes revealed that after dopamine injection, the *Ddc* level was significantly lower in the injected group, while the expression levels of *ebony* and *Bm-iAANAT* were significantly higher than those in the control group (Figure 1B). Moreover, in individuals injected with NBAD, expression of *ebony* was considerably lower than that in control pupae injected with HCl (Figure S6A). In the NADA-injected group, *Bm-iAANAT* expression was markedly lower, relative to the group injected with HCl (Figure S6B). Similar patterns were observed when NBAD and NADA were injected as controls for each other, i.e., the level of *ebony* was significantly lower in individuals injected with NBAD, while that of *Bm-iAANAT* was lower in those administered NADA (Figure S6C, D). Upon injection of catecholamines as substrates, expression levels of the genes encoding the corresponding catalytic enzymes were upregulated, and the reverse was observed when catecholamines were injected as products. The catecholamine concentration affected the expression patterns of melanin genes in injection experiments. We speculated that levels of catecholamines induce regulatory effects on the corresponding melanin genes, and analyzed the mutual regulation processes based on these interactions.

### Phenotype of the *mln* mutant after β-alanine injection

**Figure 2:**
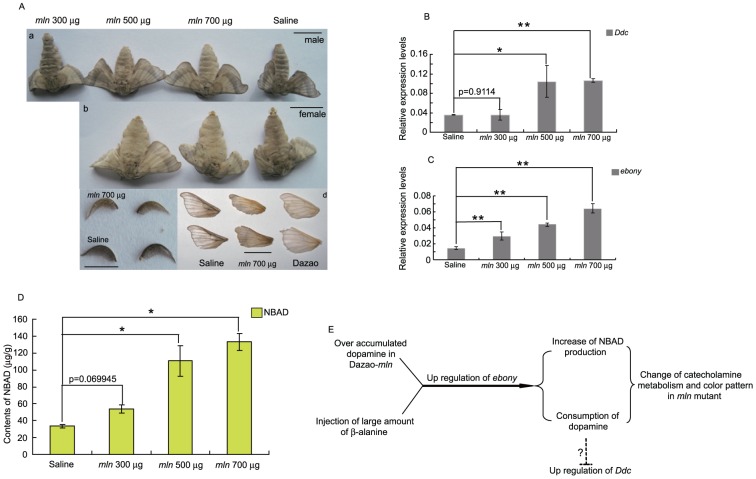
Phenotype of *mln* mutant after β-alanine injection. (A). Body color changes of *mln* subjected to different doses of injection. a and b represent the phenotypes of *mln* male and female silkworm injected with different doses, respectively. Scale bar: 1 cm; c represents color changes of tentacle at an injection dose of 700 µg/pupa. Scale bar: 5 mm; d represents color changes of vein when the injection dose is 700 µg/pupa. Scale bar: 1 cm. (B). Expression levels of *Ddc* in individuals injected with different doses of β-alanine (Student's t-test, n = 3. *p<0.05, **p<0.01, data represent mean±S.D.). (C). Expression levels of *ebony* in individuals injected with different β-alanine doses. (Student's t-test, n = 3. **p<0.01, data are presented as mean±S.D.). (D). NBAD content in individuals injected with different doses of β-alanine (Student's t-test; *p<0.05, data are presented as mean±S.D. of three separate experiments.). (E). Schematic diagram of the mechanism by which the body color of *mln* is changed after β-alanine injection. The dotted blunt symbol indicates possible decreased inhibition.

Dysfunction of the *Bm-iAANAT* gene led to dopamine over-accumulation in the *mln* mutant, resulting in a melanism phenotype [37] (Figure S3). Injection of β-alanine into mutant pupae led to its reaction with high levels of dopamine, and greater amounts of NBAD were produced, in turn, altering the make-up of catecholamines and leading to a lighter color after eclosion. The number of individuals injected and those displaying lighter body color at three different doses are listed in Table 1. Following injection of 300 µg β-alanine into pupae, no significant changes were observed among *mln* mutant moths, and only the veins of male moths showed a lighter color (Figure 2A, a). When injection doses were increased to 500 µg/pupa, 75% of individuals showed lighter overall body color, compared with the saline-injected control (Table 1. Figure 2A, a, b). At injection doses of up to 700 µg/pupa, 94% individuals exhibited lighter body color, vein and tentacles, compared to those injected with the saline control (Table 1, Figure 2A, a–d). Analysis of the corresponding melanin genes disclosed that *Ddc* was upregulated at increased injection doses, with *ebony* showing a similar regulatory pattern (Figure 2B, C). Determination of the NBAD content revealed that with increasing doses of β-alanine, more NBAD was produced, and the body color of moths became lighter and more yellow (Figure 2A, D).

**Table 1:** Doses of β-alanine injection and statistics of individual body color changes.

Dose of injection	Male	Male turned yellow	Female	Female turned yellow	Efficiency (M+F)
700 (µg/individual)	25	23	14	14	94.9%
500 (µg/individual)	20	16	12	8	75%
300 (µg/individual)	19	Not obvious	13	Not obvious	<3% (1/32)

### Catecholamine metabolism in wild-type and *mln* strains from the beginning of the fifth instar to pigmentation

**Figure 3:**
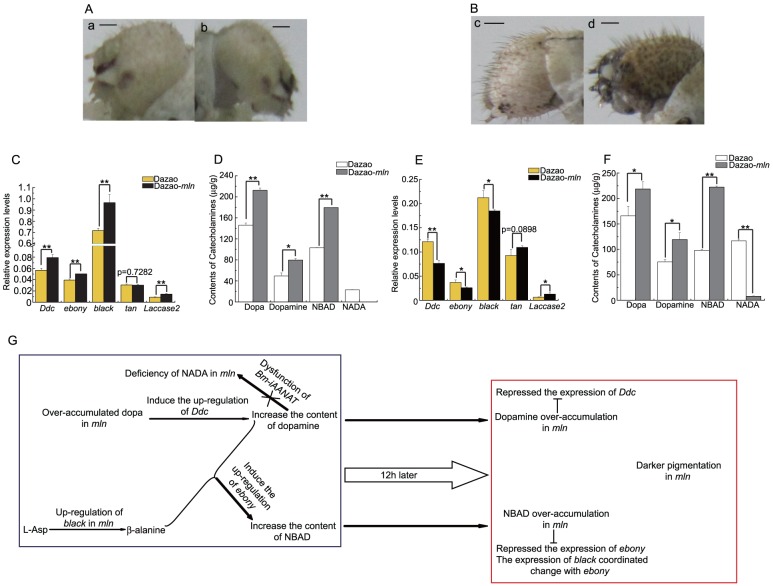
Pigmentation of head and catecholamine metabolism in fifth instar larvae after molt. (A). Color patterns of the heads from both wild-type and *mln* mutant strains immediately after the fourth molt. a and b indicate wild-type and mutant, respectively. Scale bar:1 mm. (B). Color pattern of the heads from 12 h of fifth instars of wild-type and *mln* mutant. c and d indicate the wild-type and mutant, respectively. Scale bar: 1 mm. (C). Differences in expression profiles of melanin metabolism genes in the fifth instars just after molt in both wild-type and mutant. (Student's t-test. n = 3. **p<0.01. Data are presented as mean±S.D.). (D). Differences in catecholamines between fifth instars just after molt of both wild-type and mutant. (Student's t-test. *p<0.05, **p<0.01. Data are presented as mean±S.D. of three separate experiments.). (E). Differences in the expression patterns of melanin genes between 12 h of fifth instars of both wild-type and mutant. (Student's t-test. n = 3. *p<0.05, **p<0.01). Data are presented as mean±S.D.). (F). Differences in catecholamine content at 12 h of fifth instars between wild-type and mutant strains. (Student's t-test. *p<0.05, **p<0.01. Data are presented as mean±S.D. of three separate experiments.). (G). Schematic diagram of the melanism process of the heads of mln fifth instar larva. Blue and red boxes indicate fifth instar immediately after molt and 12 h, respectively. The blunt symbol indicates inhibitory action, while the fork signifies dysfunction of the *Bm-iAANAT* gene.

The *mln* mutant silkworm exhibited melanism phenotype in the head, thoracic legs and anal plate during the larval stage. In contrast, at the beginning of each instar, these regions were not significantly different between the wild-type and mutant strains (Figure 3A, Figure. S4). Since phenotypic variation is most significantly manifested in the head, this region was selected to determine the relationship between the catecholamine content and corresponding melanin metabolism genes in both the wild-type and mutant silkworm groups. Our results showed that at the beginning of the fifth instar, the heads of both silkworm groups were not pigmented, with no notable phenotypic differences (Figure 3A). However, the dopa content in the mutant was 1.45 times that in the wild-type, while the dopamine and NBAD levels were 1.61 times and 1.74 times higher, compared to the wild-type strain, respectively (Figure 3D). However, NADA was not detectable in the mutant (Figure 3D). Expression analysis of the corresponding melanin genes showed that in the *mln* mutant, levels of *Ddc*, *ebony* and *black* genes were higher than those in the wild-type (Figure 3C). Different catecholamines display different affinities for Laccase2 protein, and Laccase2 exerts varying catalytic efficiencies for catecholamines [11], [18]. Therefore, we speculate that differences in *Laccase2* expression between mutant and wild-type strains are attributable to variations in the composition of catecholamines. In other words, at the beginning of the fifth instar, higher levels of dopamine and NBAD provided the materials for the darker body color of the mutant strain. The heads of both the *mln* mutant and wild-type silkworm were pigmented at 12 h of the fifth instar, and mutant heads were clearly melanized. At this stage, the mutant contained slightly more dopa than wild-type (Figure 3F). The dopamine content in the mutant was 1.58 times, while the NBAD content was 2.27 times that of wild-type. The abundance of these two catecholamines corresponded with the dark brown phenotypes of the mutant head. The NADA level in the mutant was markedly lower than that in the wild-type, suggesting dysfunction of the *Bm-iAANAT* gene (Figure 3F). Analysis of the corresponding melanin genes disclosed that in the mutant strain, the expression levels of *Ddc*, *ebony* and *black* were significantly lower those in the wild-type, while that of *tan* (NBAD hydrolysis gene)was slightly higher (Figure 3E). *Laccase2* expression was additionally higher in the mutant strain (Figure 3E).

### Analysis of similar pigmentation between wild-type and *mln* pupae during the early stages of pupation

**Figure 4:**
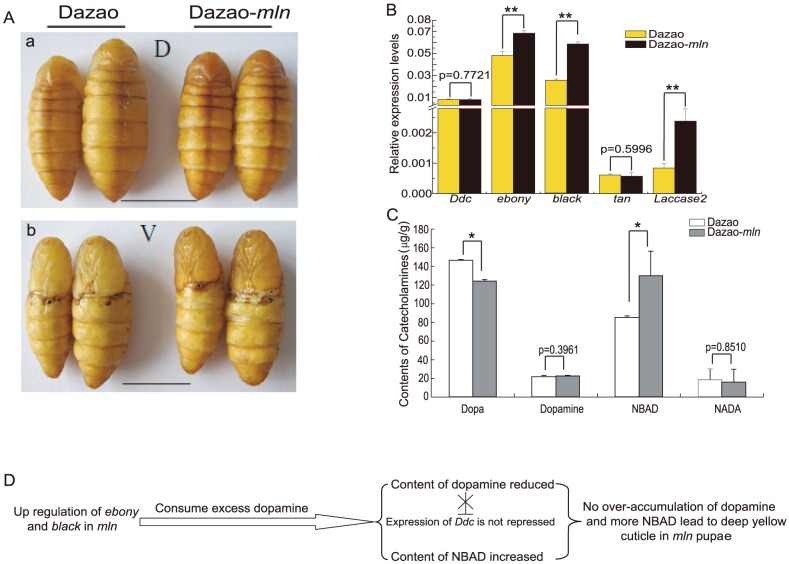
Mechanism underlying the phenotypic similarity between the wild-type and mutant silkworm during early stages of pupation. (A). Phenotypic similarities between the wild-type and mutant at day 2 of pupation. a and b indicate phenotype of the dorsal (D) and ventral side (V) of the wild-type and mutant strains, respectively. Scale bar: 1 cm. (B). Expression differences in melanin metabolism genes between wild-type and mutant pupae at day 2 of pupation. (Student's t-test. n = 3. **p<0.01. Data are presented as mean±S.D.). (C). Differences in catecholamine contents between wild-type and mutant pupae at day 2 of pupation. (Student's t-test. *p<0.05. Data are presented as mean±S.D. of three separate experiments.). (D). Schematic diagram of the mechanism attributing to similar phenotypes between wild-type and mutant silkworm during early stages of pupation. A blunt symbol with a fork indicates that substantial consumption of dopamine releases *Ddc* inhibition in the mutant.

The melanic mutants *sooty* (caused by dysfunction of *Bmebony*, leading to dopamine over-accumulation and melanization) and *black pupa* (caused by disruption of the regulatory sequences of *Bmblack*, resulting in its downregulation and lack of β-alanine, compared with wild-type, which, in turn, causes insufficient production of NBAD) have been identified in silkworm pupa. The *black pupa* (*bp*)mutation is caused by over-accumulation of dopamine at the early stages of pupation (unpublished data), and manifests as a melanism phenotype characterized by high levels of dopamine and its subsequent oxidation to melanin after transportation out of the cell. Moreover, this finding indicates that at the early stages of pupation, expression levels of *ebony* and *black* are crucial for pigmentation (to determine body color). After administration of dopamine into early-stage Dazao-*mln* pupae (injected at days 2 and 4 of pupation and examined on days 3 and 5, respectively), body color became darker with a tendency to develop stronger melanization (Figure S5). Following injection, melanization became more significant with time, but we observed no evidence of a regulatory point at which melanin precursors are controlled. This indicates that at early stages of pupation, transport of melanin precursors out of the cell is not the crucial factor for melanin deposition. In other words melanin deposition is not determined by the exit mechanism or receptors, but controlled by the regulation of pigment genes. High or abnormal expression of *ebony* and *black* appear critical for pupa body color regulation, since these genes are linked to the consumption or conversion of dopamine. This intriguing scenario in which *mln* is not melanized at early stages of pupation and resembles the wild-type phenotype (Figure 4A) suggests that in the mutant, over-accumulated dopamine resulting from dysfunction of *Bm-iAANAT* may be consumed via the regulation of related pigment genes, and is most likely converted into NBAD, a precursor for yellowish pigments.

To elucidate the underlying mechanism, pupae at day 2 of pupation (P2) from both wild-type and mutant silkworms were selected (pupa was pigmented at this time, and this was the first stage where differences in pigmentation between the wild-type and mutant could be observed). The results of catecholamine determination showed that the dopamine levels were similar between the wild-type and mutant strains, whereas the NBAD level was 51.6% higher in the *mln* mutant (Figure 4C). Moreover, the NADA content was extremely low in both wild-type and mutant strains (Figure 4C), similar to levels in the pupa of tobacco hornworm, *manduca sexta* [30]. Simultaneously, the expression of *Bm-iAANAT* reported previously was significantly lower at the wandering and early pupal stages (W0-P2), compared to the late pupation stage (P7–P8) [37], indicating that NADA does not represent the main tanning material during the early pupation period. Real-time RT-PCR analysis of melanin metabolism genes revealed no significant differences in the expression levels of *Ddc* between the wild-type and mutant silkworm strains, indicating that *Ddc* is not suppressed in the mutant and dopamine does not accumulate (Figure 4B). However, expression levels of *ebony* and *black* were upregulated in the mutant, compared with wild-type. Accordingly, we speculate that in the *mln* mutant, owing to the upregulation of *ebony* and *black*, excess dopamine that accumulates during the fifth instar larva stage is consumed, in turn, releasing the repression effect on *Ddc* and generating NBAD (Figure 4D). Thus, in the early stages of pupation, normal amounts of dopamine and high levels of NBAD lead to a more yellowish body color in the *mln* mutant (Figure 4D).

### Differences in catecholamine metabolism between wild-type and *mln* at the moth stage

**Figure 5:**
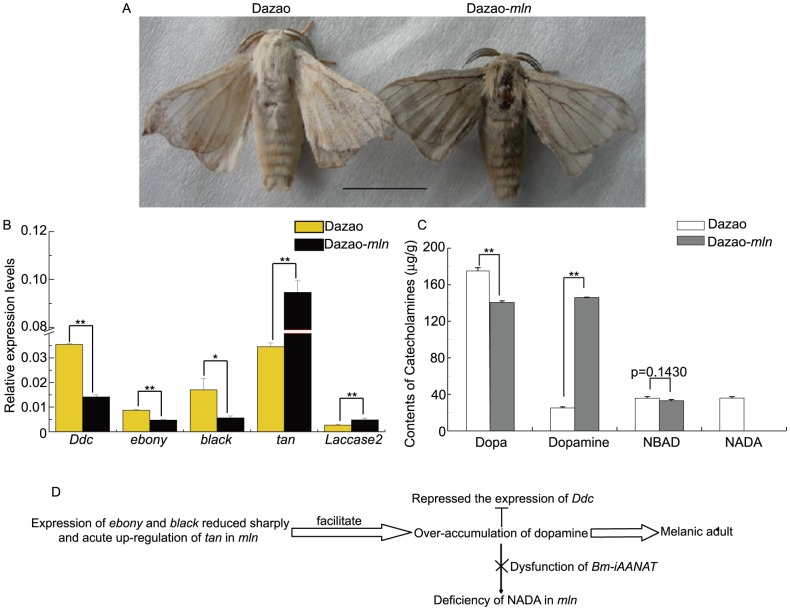
Analysis of phenotypic differences between wild-type and mutant adult silkworm. (A). Phenotype of *mln* and wild-type adults. Scale bar: 1 cm. (B). Differences in expression patterns of pigment metabolism genes between *mln* and wild-type adults. (Student's t-test. n = 3. *p<0.05, **p<0.01. Data are presented as mean±S.D.). (C). Differences in catecholamine content between *mln* and wild-type adults (Student's t-test. **p<0.01. Data are presented as mean±S.D. of three separate experiments.). (D). Schematic diagram of the melanism process in the *mln* adult. The Blunt symbol indicates inhibitory action, while the fork signifies dysfunction of the *Bm-iAANAT* gene.

The whole body of adult *mln* mutant was melanized, while wild-type Dazao exhibited white scales and yellow exoskeleton (Figure 5A). Investigation of catecholamines in wild-type and mutant silkworms at this stage revealed lower amounts of dopa in the mutant (∼81% that in the wild-type; Figure 5C), which correlates well with our previous finding that expression of *pale* in the mutant was 79% that in the wild-type during the moth stage [37]. However, significantly higher quantities of dopamine (∼5.83 times that in wild-type; Figure 5C), were observed in the mutant strain. Simultaneously, *Ddc* expression was lower in the mutant, suggesting an inhibitory effect of over-accumulated dopamine, in keeping with our previous report (Figure 5B) [37]. No NADA was detected in the mutant, which correlated well with dysfunction of the *Bm-iAANAT* gene (Figure 5C). The NBAD content was not significantly different between the mutant and wild-type strains. However, *ebony* and *black*, two genes involved in NBAD production, were expressed at higher levels in the wild-type (Figure 5B). Interestingly, the *tan* gene responsible for converting NBAD to dopamine was expressed at significantly higher levels in the mutant (Figure 5B). Moreover, the *Laccase2* level was higher in the mutant than wild-type (Figure 5B). We hypothesized that downregulation of *black* and *ebony*, coupled with upregulation of *tan*, facilitated dopamine accumulation (Figure 5D). Meanwhile, dysfunction of *Bm-iAANAT* meant that dopamine could not be converted into NADA, and therefore, the dopamine content in adult *mln* was significantly higher compared to its wild-type counterpart. This, in turn, repressed *Ddc* gene expression and generated the melanic phenotype (Figure 5D).

### Dynamic changes in phenotype and melanin gene expression patterns during different stages from pupa to moth between wild-type and *mln* silkworm

**Figure 6:**
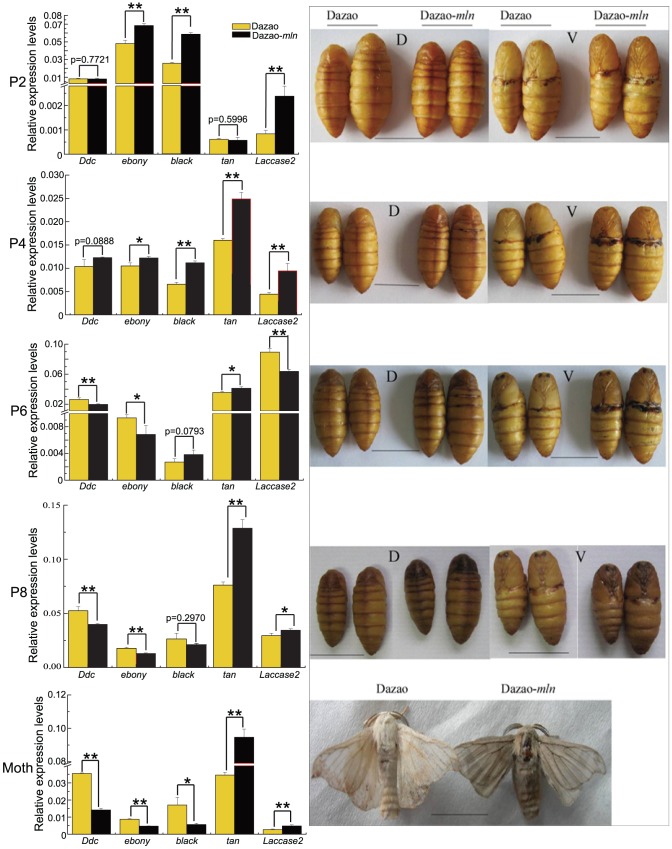
Phenotypes and expression levels of melanin genes between *mln* and wild-type from pupal to moth stages. P2, P4, P6 and P8 indicate days 2, 4, 6 and 8 of pupation, respectively. D and V represent dorsal side and ventral side of pupa. Scale bar: 1 cm. (Student's t-test. n = 3. *p<0.05, **p<0.01. Data are presented as mean±S.D.).

We selected days 4 (early-middle stage; P4), 6 (middle stage; P6) and 8 (late-stage P8) pupae of both wild-type and mutant strains to investigate the differences in expression of melanin metabolism genes. These findings, coupled with those obtained from pupae at day 2 of pupation (specified previously) and the expression profile of melanin synthesis genes at the moth stage (specified previously), were used for comparative analysis of dynamic changes of these genes from the pupa to moth stage. The ratios of the expression levels of these genes in the mutant and wild-type strains (Dazao-*mln*/Dazao) are listed in Table 2. Expression levels of *Ddc* in the mutant at early pupa (P2) and early-middle pupa (P4) stages were similar to or higher than those in the wild-type strain. Concurrently, expression levels of *ebony* and *black* were higher in the mutant than wild-type silkworm during this period, and *mln* pupa was more yellow than wild-type pupa (Table 2, Figure 6). From the middle stage of pupa (P6) to ecdysis (moth stage), expression levels of *Ddc* in the *mln* mutant were markedly lower than in wild-type, and the ratios of *ebony* and *black* expression levels to their counterparts in wild-type also decreased with time (Table 2, Figure 6). The ratio of *ebony* was altered from 0.73 at P6 to 0.54, and *black* from 1.42 at P6 to 0.33 at the moth stage (Table 2). Moreover, melanization was initiated during the middle stage of pupa (P6) in the mutant. Expression of *tan* in the mutant strain was upregulated relative to that in wild-type from the late stage of pupa (pupa of *mln* was notably melanized at the late stage) (Table 2, Figure 6). Expression of *Laccase2* was consistently higher in the mutant than wild-type, except at day 6 of pupation (P6) (Table 2, Figure 6).

**Table 2:** *Ratios of melanin gene expression levels during different developmental stages.

	Development stages				
Genes	P2	P4	P6	P8	Moth
***Ddc***	0.977524	1.183614	0.749097	0.760089	0.398298
***ebony***	1.421761	1.163378	0.734871	0.726879	0.538417
***black***	2.275977	1.707544	1.415743	0.798224	0.328333
***tan***	0.932897	1.554360	1.159594	1.690904	2.738983
***Laccase2***	2.836710	2.124887	0.713391	1.201665	1.784255

* **(Ratio = (mean of quantitative PCR for Dazao-** ***mln*** **)/(mean of quantitative PCR for Dazao)).**

### Scanning electron microscope analysis of dorsal plate sections from wild-type and *mln* strains

**Figure 7:**
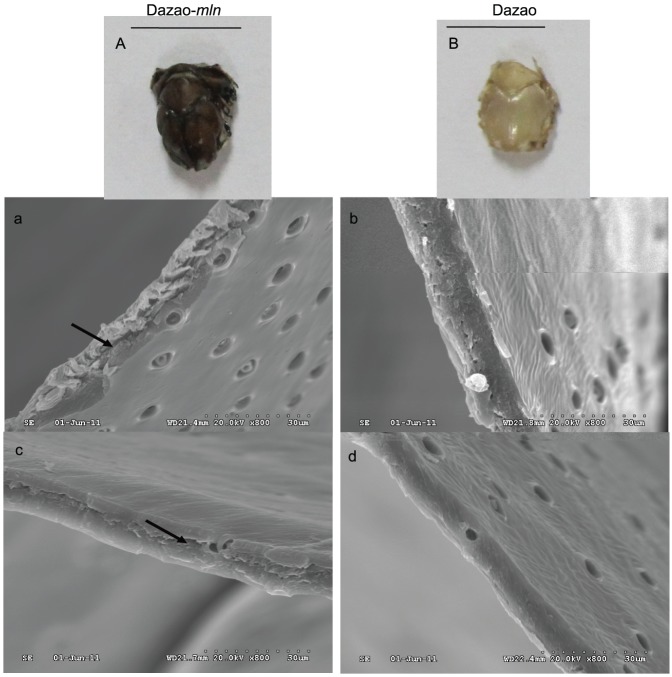
Scanning electron microscope analysis of a cross-section of dorsal plates from wild-type and *mln* strains. A and B represent adult dorsal plates of *mln* and wild-type silkmoths, respectively. Scale bar: 5 mm. a and c are cross sections of the adult dorsal plate of *mln*. The arrow indicates stratification. b and d represents cross-sections of the adult dorsal plate of Dazao. Magnification was described as X.

Scanning electron microscope analysis revealed that the dorsal plate section of Dazao was even and dense, while crevices existed in the dorsal plate of the *mln* mutant (Figure 7). Stratification was additionally observed in the mutant (Figure 7). After injection of NBAD into the *mln* mutant, dorsal plates of injected individuals were more yellow in color than those not injected, demonstrating greater NBAD sclerotin deposition in the dorsal plates (Figure S7). Moreover, no stratification was observed in the dorsal plates of injected individuals, verifying that NBAD acts as a cross-linking agent in the exoskeleton and indirectly demonstrating that the catecholamine does not exist at levels higher than those required (otherwise the dorsal plate of the *mln* mutant would not be stratified) (Figure S7). Moreover, the dorsal plates of the *mln* individuals injected with NADA displayed similar properties to those injected with NBAD. Specifically, stratification was evidently eased, and the dorsal plates became denser (Figure S7). In view of the deficiency of NADA in the *mln* mutant (Figure 5C) and its function in the cross-linking of insect cuticles [5], [26], [42], we concluded that the formation of different layers in the *mln* mutant is closely related to the lack of this catecholamine.

### Mechanical properties of adult anterior wings in wild-type and *mln* silkworm

**Figure 8:**
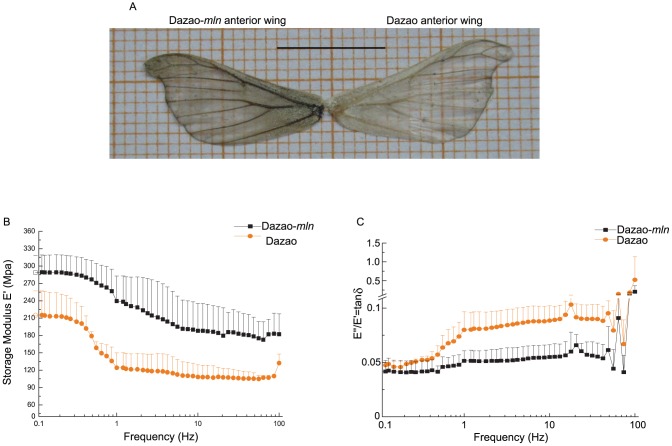
Mechanical properties of adult wings between wild-type and *mln* strains. (A). Phenotype of the anterior wing in wild-type and *mln* mutant. (B). Storage modulus (E′) of adult wings of wild-type and *mln* mutant under frequency scanning (n = 3). (C). tanδ(E″/E′) of adult wings of wild-type and *mln* mutant under frequency scanning (n = 3).

Metabolic differences in catecholamines affect not only insect body color but also the tanning process of the epidermis. During tanning, oxidized products of catecholamines cross-linked with cuticle proteins play important roles in the physical properties of insects, such as hardness, stiffness, cross-linking and mechanical properties [18], [35]. The dynamic mechanical properties of material are evaluated based on various factors, such as Modulus of elasticity (E′), presented as the ratio of normal stress to normal strain within the elastic deformation stage of the material, Loss modulus (E″), representing energy loss caused by viscous deformation when the material is deformed, Loss tangent (E″/E′ ratio, termed ‘mechanical loss factor’), represented by tanδ, assessed via dynamic mechanical analysis (DMA) that provides information on the inner molecular structures [43], [44]. We investigated the mechanical properties of the wings from both wild-type and mutant silkworm (color patterns of anterior wings were consistent with their respective body color patterns, and differences in melanin gene expression between wings of specimens from both strains were similar to those found between moths of wild-type and mutant strains (Figure 8A, Figure S8) and analyzed the effects of catecholamines on wing mechanical properties using a dynamic mechanical analyzer (DMA Q800). Our results showed that the modulus of elasticity (E′) of both types of wings increased with a decrease in frequency, and E′ of mutant wings was higher than that of wild-type wings at all times (Figure 8B). However, the loss factor (tanδ) for wings from both strains was reduced with the decrease in frequency, and tanδ of wild-type was consistently higher than that of the mutant group (Figure 8C).

## Discussion

We observed that after dopamine injection (P6), *mln* pupa was melanized via a gradual darkening process, indicating that the precursors for melanization accumulated slowly. Thus, during a specific time-period, all precursors existed in excess amounts or accumulated in cells (Figure 1A). Investigation of the expression levels of the corresponding genes revealed that the dopamine synthase gene, *Ddc*, was downregulated, compared with control, while genes encoding proteins that catalyze dopamine, *ebony* and *Bm-iAANAT* were evidently upregulated (interestingly, although the Bm-iAANAT protein is defective in *mln*, expression of its corresponding gene was upregulated). In contrast, the *Bm-iAANAT* level was markedly lower in the NADA-injected group than control. Although the structure of the *Bm-iAANAT* gene is defective (the defect is not at the 5′ regulatory zone of the gene) and cannot encode functional enzyme in *mln*, injection of excess NADA or dopamine into the *mln* mutant affected the expression of the *Bm-iAANAT* gene (Figure 1B, Figure S6). The issue of whether this regulation bypasses the influence of the substrate on its enzyme and directly affects DNA is yet to be established, and will be further investigated in our follow-up research. Based on the results obtained from injection experiments, we propose that the substrate acts upon genes encoding enzyme products (for example, when catecholamines are over-accumulated, the expression levels of their corresponding genes are downregulated). We suggest that materials used for synthesis of these functional enzymes are valuable for organisms and not wasted. When a particular material exists in large amounts in an organism, production of large quantities is no longer required and generation of the enzyme that catalyzes its synthesis should be slower, therefore avoiding wastage and ensuring that materials are utilized economically. Accordingly, expression of genes encoding the enzyme should be downregulated, thereby reducing enzyme production. Thus, downregulation of *Ddc* observed during melanization may be explained as follows: when dopamine is over-accumulated, the DDC enzyme is no longer needed, leading to the lowering of transcription, in turn, reducing enzyme synthesis and saving material. In other words, when the product accumulates, the total amount of synthase may remain unchanged, but its rate of increase is significantly lower. When the concentration of one substrate is higher, the gene encoding the corresponding enzyme will be upregulated (termed ‘upregulation induced by substrate’).

In some melanism mutants of insects, upon injection of β-alanine, the dopamine that accumulates is consumed, restoring the phenotype to wild-type (yellow or amber) [13], [18]. In the current study, after β-alanine injection, *ebony* expression was upregulated in the injected individuals, and the amount of NBAD, a precursor for yellow pigment, was increased (Figure 2C, D). We propose that large amounts of β-alanine coupled with accumulating dopamine induce *ebony* expression, in turn, leading to greater NBAD production, causing the *mln* mutant to become yellower. Changes in catecholamine metabolism in the injected group (more active from dopamine to NBAD) induced alterations in their pigmentation patterns. Moreover, after the β-alanine dose was increased, *Ddc* was upregulated in the injected individuals. In view of this result, combined with the expression data obtained for melanin genes after catecholamine injection in the *mln* mutant, we proposed that accumulated dopamine (product) represses the increase in DDC enzyme, causing its synthesis to slow, represented by a decrease of transcription. Following injection of β-alanine, over-accumulated dopamine is consumed (converted into NBAD). This reduction in its total level may cause a decrease in its inhibitory effect on DDC enzyme, leading to increased *Ddc* gene expression. Another possible explanation is that after injection of excess β-alanine, conversion of dopamine to NBAD is sped up, which increases the demand for dopamine and enhances the expression levels of *Ddc*. This ensures that sufficient dopamine is converted into NBAD (the amount of total dopamine may vary only slightly, but the production and consumption rates are increased). We will continue to investigate this theory in follow-up research.

We speculate that at the beginning of the fifth instar, over-accumulated dopa (the underlying reason for dopa accumulation is yet to be established and will be investigated in future studies) induces upregulation of *Ddc* in the *mln* mutant, which, coupled with dysfunctional *Bm-iAANAT*, results in the rapid accumulation of dopamine (Figure 3C, D and G). The high level of dopamine may induce upregulation of *ebony*, in turn, triggering consumption of more β-alanine, and leading to upregulation of *black* and eventual production of more NBAD in the mutant (Figure 3C, D and G). We assume that this is the stage where pigments begin to increase and accumulate, and therefore, the high levels of pigment precursors in the mutant at this time do not suppress melanin metabolism gene expression. Furthermore, at this time, the total catecholamine content in the mutant strain is higher than that in the wild-type strain. Differences in the make-up and content of catecholamines between the *mln* mutant and wild-type provide the basis for phenotypic variations. Pigmentation is already underway at 12 h of the fifth instar, and differently pigmented heads of the mutant and wild-type indicate that the catecholamines preserved in epidermis cells are converted to colored quinones, which are mixed into the cuticle matrix. Although the dopamine content is still higher in the mutant during this time, *Ddc* expression is lower in the wild-type strain. We propose that this is due to the repressional effect of over-accumulated dopamine in the mutant (from 79.625±2.892 µg/g to 119.315±13.683 µg/g) (Figure 3D, F and G). Moreover, accumulation of NBAD (from 179.625±0.134 µg/g to 222.380±1.881 µg/g) in the mutant led to suppression of *ebony* and *black* expression, resulting in lower levels than wild-type (Figure 3D, F and G). Accumulation of NBAD also triggered expression of *tan*, leading to slightly higher expression in the mutant strain. Thus, *tan* is expressed at a higher level in *mln*, implying that accumulating NBAD is converted to dopamine, once again leading to the over-accumulation of dopamine. To sum up, at this stage, more total catecholamines (melanin precursors) still exist in the mutant. Thus, during this period, over-accumulation of dopamine and NBAD lead to darkening of pigment in the *mln* mutant (Figure 3G). In fact, from the beginning to 12 h of fifth instar, phenotypic differences between the mutant and wild-type are initiated, while the significant differences in catecholamines and their related genes are already established. During the larval stage, the phenotypic differences between the two silkworm strains become more evident with time. We will further focus on how pigments accumulate and expression of their corresponding genes affect the subsequent melanization step in the mutant.

**Figure 9:**
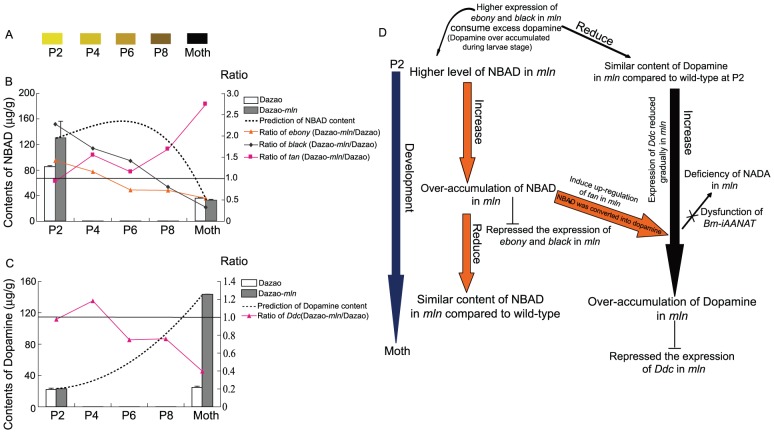
Schematic diagram of variations in dopamine and NBAD contents of *mln* mutant from the pupal to moth stage. (A). Color pattern changes from pupa to moth in *mln*. (B). Variations in the ratios of *ebony*, *black* and *tan* from pupa to moth stages and the speculated NBAD content variation diagram. Ratio = (mean of quantitative PCR for Dazao-*mln*)/(mean of quantitative PCR for Dazao)). (C). Variations in the ratio of *Ddc* from pupal to moth stage and speculated dopamine content variation diagram. Ratio = (mean of quantitative PCR for Dazao-*mln*)/(mean of quantitative PCR for Dazao)). (D). Schematic diagram of the melanization of *mln* from pupa to moth stage. The circular arrow indicates that high expression of *ebony* and *black* promotes production of NBAD in the mutant. The blunt symbol indicates inhibitory action, while the fork signifies loss of *Bm-iAANAT* gene function.

During the early stages of pupation, the dopamine content in *mln* is similar to that in wild-type, while the NBAD content is higher in the mutant (Figure 4C). In contrast, at the moth stage, the dopamine content in *mln* is significantly higher than in wild-type, while that of NBAD remains similar in both strains (Figure 5C). In the *mln* mutant, the means by which corresponding genes regulate the dopamine and NBAD levels during development from pupa to moth to make the pigment remains unclear at present. Based on the expression levels of the corresponding melanin genes in the wild-type and mutant strains from P2 to moth stage (Table 2, Figure 6), we speculate that between P2 and P4, the upregulated *ebony* and *black* genes consume dopamine and produce more NBAD in the mutant (Table 2, Figure 9A, B, Figure 6). Thus, during this period, the *mln* mutant color appears more yellow than the wild-type silkworm (Figure 9A, B, Figure 6). Based on the ratios of *ebony* and *black* in the mutant to wild-type from P2 to P6 and NBAD contents at P2 (Table 2. Figure 4C, Figure 6), we speculate that increased levels of *ebony* and *black* from P2 to P4 and high expression of *black* at P6 lead to over-accumulation of NBAD in *mln*, and the accumulation rates in the early/early-to-middle stages of pupation (P2 to P4) are higher than those in the middle stages of pupation (P6) (Figure 9B). From day 8 of pupation to the moth stage, *ebony* and *black* are significantly downregulated owing to the repressional effect of NBAD, and excess NBAD promotes acute upregulation of *tan*, leading to conversion into dopamine (Table 2, Figure 9B, D, Figure 6). *Ddc* expression in the mutant during the early/early-middle stages of pupa (P2, P4) is not lower than that in the wild-type, indicating that dopamine does not repress *Ddc*. Concurrently, the mutant pupa is not melanized during this period, signifying that dopamine does not accumulate excessively in the mutant. (Table 2, Figure 9C, Figure 6). However, the ratio of *Ddc* is evidently lower during the middle (P6) and late pupal stages (P8) (Table 2, Figure 9C). At this time, the mutant body color becomes notably darker, and melanization is initiated (Figure 6). This finding implies that dopamine accumulates during these stages. Moreover *Bm-iAANAT* is highly expressed during this period, (observed from our previous research). Owing to dysfunction of *Bm-iAANAT*, the mutant cannot consume excess dopamine, and therefore, the dopamine content becomes higher with time. At the moth stage, the *Ddc* ratio is only 0.398, whereas the dopamine level is considerably higher in the mutant (Figure 5C). Based on the dynamic expression changes of *Ddc* in the *mln* mutant, we propose the accumulation of dopamine begins at the middle stages of pupa (from P6) (Figure 9C). On the one hand this over-accumulation is caused by degradation of NBAD, and on the other, by dysfunction of the *Bm-iAANAT* gene (Figure 9D).

Notably, in the mutant strain, from the middle and late stages of pupa to the moth stage, low expression of *ebony* and high expression of *tan* facilitate melanization, which correlates well with the earlier finding that downregulation of *ebony* and upregulation of *tan* lead to darker body color in *drosophila melanogaster* [45]. In the mutant, the massive dopamine accumulation caused by dysfunction of *Bm-iAANAT* and degradation of NBAD does not lead to upregulation of *ebony*, and in the injection experiment, only high doses of β-alanine (≥500 µg/pupa) can induce *ebony* and consumption of excess dopamine. Accordingly, we speculate that the catecholamine content and regulation of the corresponding genes trigger conversion of NBAD into dopamine and dopamine melanin, which form the dominant pathway from the middle/late pupa to moth stage in the mutant strain. We will further test this hypothesis during follow-up research.

The dynamic mechanical properties of film-like materials can be determined based on the stretching mode of DMA [46], [47]. The moth wings are relatively wide and thin, and can thus be treated as film-like material to analyze their mechanical properties in both wild-type and mutant strains. The wings of insects provide survival advantages in finding food, mate choice, migration, and predation [48]. Therefore, information on the mechanical properties of differentially pigmented wings could further our understanding of the influence of catecholamines on survival and evolution of insects. In mechanical analyses, the stretch test was carried out using a range of high (100 Hz) to low (0.1 Hz) frequencies, since the action time is lower with higher frequencies. Over a certain time-period when the possibility of wings being torn apart exists, testing a range of frequencies has an advantage, as high frequencies provide more data. In DMA analysis, Storage modulus (E′) provides a measurement of material stiffness [18], [35], [43], [44], [49]. Our results showed that E′ of the mutant is higher than that of the wild-type strain, indicating that the wings of the mutant are stiffer (Figure 8B). The damping characteristics of the material are represented by tanδ [18], [35], [43], [44], [49]. Higher tanδ observed for wild-type signifies that the damping characteristics of the wild-type are better than those of the mutant strain (Figure 8C). Materials with better damping characteristics can dissipate energy absorbed in the form of heat, therefore reducing their amplitude, which results in damping of oscillation [44]. From the above results, we propose that the wings of wild-type silkworm are better at damping oscillation, compared with those of the mutant, when subjected to outside forces. Moreover, proper make-up of catecholamines can endow wings with higher storage modulus and damping factor, which facilitates better adaptation to the environment. The pigment pattern (color and expression patterns of the corresponding melanin genes) of the wings of the *mln* mutant is similar to that of wild-type at the moth stage and the near-isogenic line, Dazao-*mln*, is back-crossed for 24 generations. Thus, theoretically, the majority of its genome is identical to its recurrent parent, Dazao, and the differences between the wild-type and *mln* mutant are mainly attributable to variations in the catecholamine content caused by abnormality of the *Bm-iAANAT* gene in the mutant strain. Accordingly, we propose that the differences in mechanical properties between *mln* and wild-type are closely related to the abnormal catecholamine metabolism in *mln* (caused by dysfunction of the *Bm-iAANAT* gene). Furthermore, the hardness of the sclerotized regions may be a hurdle for *mln* moths during copulation (the genitalia of the *mln* mutant strain are darker and harder than wild-type, as observed by Professor Toru Shimada and our group; Figure S9).

In summary, experimental data obtained by our group using *mln* silkworm as a model reveal that the metabolism of catecholamines and regulation of the corresponding melanin metabolism genes play an important role in determining body color and cuticle physical properties of lepidoptera insects. Metabolic changes of catecholamines in insects may be associated with behavior, such as gregation,locomotion, learning and homosexuality [50], [51], [52], [53], [54]. We have additionally observed differences in locomotion and homosexuality of males between the wild-type and *mln* mutant strains (unpublished data). Future research will focus on the influence of catecholamines on the behavior of lepidoptera insects and the underlying molecular mechanisms.

## Supporting Information

Figure S1 **Schematic diagram of melanin metabolism pathway in silkworm and corresponding enzyme mutant.** PO, phenol oxidase; YELLOW, major royal jelly protein; ADC, Aspartate decarboxylase, DDC, dopa decarboxylase; EBONY, N-β-alanyl dopamine synthetase; TH, tyrosine hydroxylase. TAN, N-β-alanyl dopamine hydrolysis enzyme. The pigment precursors are shown in blue. The enzymes are shown in red. Publisher's and Author's consent have been obtained for using pictures of these mutants.
(EPS)

Figure S2 **HPLC analysis of standard samples of four kinds of catecholamines.** Arrows indicate the peaks of four standard samples. Peaks in the box are peaks of the content of the solution that used to dissolve samples.
(EPS)

Figure S3 **RNAi of** ***Bm-iAANAT*** **gene.** (A). Phenotype of RNAi individuals. a and b are phenotype of female moth and male moth, respectively. Scale bar: 1 cm. (B) Statistics of RNAi. (C) Detection of *Bm-iAANAT* expression levels. (Student's t-test. n = 3. **p<0.01, The data show the mean±S.D.). Four siRNA fragments were designed and synthesized by Guangzhou RiboBio Co.,LTD to carry out RNAi. DNA sequences of templates for siRNA are listed in supplementary data. Four fragments were mixed at a 1∶1∶1∶1 ratio, and injected at 1 µg/pupa, 2 µg/pupa and 3 µg/pupa at day 1, 4 and 6 of pupation, respectively. siRNA of GFP were used as control, with same injection dose and period as the experimental group.
(EPS)

Figure S4 **Phenotype of thoracic legs and anal plate in wild-type and** ***mln*** **mutant just after the fourth molt.** Scale bar: 2 mm.
(EPS)

Figure S5 **Phenotype analysis of Dazao-** ***mln*** **pupae after dopamine injection.** A is P3 pupae after injected with dopamine at P2 (the degree of melanization become greater as time goes on); B is P5 pupae after injected with dopamine at P4 (the degree of melanization become greater as time goes on). D: dorsal view. V: ventral view. Scale bar: 1 cm.
(EPS)

Figure S6 **Relative Expression levels of melanin genes in Dazao-** ***mln*** **after injection of NBAD and NADA at P6 (P7 pupae were used for investigation).** n = 3. Student's t test.* represents p<0.05, **represents p<0.01. A: Differences in *ebony* expression between individuals injected with NBAD and individuals injected with HCl as control. B: Differences in *Bm-iAANAT* expression between individuals injected with NADA and individuals injected with HCl as control. C: Differences in *ebony* expression between individuals injected with NBAD and individuals injected with NADA. D: Differences in *Bm-iAANAT* expression between individuals injected with NADA and individuals injected with NBAD.
(EPS)

Figure S7 **Scanning electron microscopy analysis of cross-section of dorsal plates from wild-type, Dazao-** ***mln*** **, Dazao-mln injected with NBAD and Dazao-** ***mln*** **injected with NADA.** A and B are dorsal plates of wild-type and Dazao-*mln*, respectively. C and D are dorsal plates of Dazao-*mln* injected with NBAD and Dazao-*mln* injected with NADA, respectively. Scale bar: 5 mm. Arrowhead indicate stratification. Magnification is represented by X.
(EPS)

Figure S8 **Investigation of relative expression levels of corresponding melanin genes in anterior wings between Dazao-** ***mln*** **and wild-type.** n = 3. student's t test. **represents p<0.01.
(EPS)

Figure S9 **Phenotype of genitalia between Dazao-** ***mln*** **and wild-type.** A: female genitalia. B: male genitalia. Scale bar: 0.5 cm.
(EPS)

Table S1 **The primers for Quantitative RT-PCR and the template DNA sequences of siRNA.**
(XLS)
